# Analysis of the Targets and Glycosylation of Monoclonal IgAs From MGUS and Myeloma Patients

**DOI:** 10.3389/fimmu.2020.00854

**Published:** 2020-05-27

**Authors:** Adrien Bosseboeuf, Célia Seillier, Nicolas Mennesson, Sophie Allain-Maillet, Maeva Fourny, Anne Tallet, Eric Piver, Philippe Lehours, Francis Mégraud, Laureline Berthelot, Jean Harb, Edith Bigot-Corbel, Sylvie Hermouet

**Affiliations:** ^1^CRCINA, Inserm, Université de Nantes, Université d'Angers, Nantes, France; ^2^Laboratoire de Biochimie, CHU de Tours, Tours, France; ^3^Inserm UMR966, Tours, France; ^4^Inserm U1053, Université de Bordeaux, Bordeaux, France; ^5^Laboratoire de Bactériologie, Centre National de Reference des Campylobacters et des Hélicobacters, CHU de Bordeaux, Bordeaux, France; ^6^Centre de Recherche en Transplantation et Immunologie UMR1064, Inserm, Université de Nantes, Nantes, France; ^7^Laboratoire de Biochimie, CHU de Nantes, Nantes, France; ^8^Laboratoire d'Hématologie, CHU de Nantes, Nantes, France

**Keywords:** monoclonal immunoglobulin A (IgA), multiple myeloma, monoclonal gammopathy of undetermined significance (MGUS), infectious antigens, Epstein–Barr virus, hepatitis C virus, lysoglucosylceramide (LGL-1), sialylation

## Abstract

Previous studies showed that monoclonal immunoglobulins G (IgGs) of “monoclonal gammopathy of undetermined significance” (MGUS) and myeloma were hyposialylated, thus presumably pro-inflammatory, and for about half of patients, the target of the monoclonal IgG was either a virus—Epstein–Barr virus (EBV), other herpes viruses, hepatitis C virus (HCV)—or a glucolipid, lysoglucosylceramide (LGL1), suggesting antigen-driven disease in these patients. In the present study, we show that monoclonal IgAs share these characteristics. We collected 35 sera of patients with a monoclonal IgA (6 MGUS, 29 myeloma), and we were able to purify 25 of the 35 monoclonal IgAs (6 MGUS, 19 myeloma). Monoclonal IgAs from MGUS and myeloma patients were significantly less sialylated than IgAs from healthy volunteers. When purified monoclonal IgAs were tested against infectious pathogens and LGL1, five myeloma patients had a monoclonal IgA that specifically recognized viral proteins: the core protein of HCV in one case, EBV nuclear antigen 1 (EBNA-1) in four cases (21.1% of IgA myeloma). Monoclonal IgAs from three myeloma patients reacted against LGL1. In summary, monoclonal IgAs are hyposialylated and as described for IgG myeloma, significant subsets (8/19, or 42%) of patients with IgA myeloma may have viral or self (LGL1) antigen-driven disease.

## Introduction

Myeloma is preceded by an asymptomatic stage termed monoclonal gammopathy of undetermined significance (MGUS) (1–4). In MGUS and myeloma, clonal plasma cells produce large quantities of a so-called “monoclonal” immunoglobulin (Ig). In MGUS, clonal plasma cells represent <10% of bone marrow cells, and the amount of monoclonal Ig in blood is <30 g/L. Following the acquisition of genetic alterations in clonal plasma cells, a fraction of MGUS progress over time toward smoldering, then overt myeloma (5, 6). The rate of transformation of MGUS toward myeloma is 1% per year per individual. In myeloma, patients suffer from diverse renal, bone, and hematological complications; clonal plasma cells represent >10% of bone marrow cells, and the amount of monoclonal Ig in blood is >30 g/L (6). For 55–60% of MGUS and myeloma patients, the monoclonal Ig is type G, for 20–25%, it is type A, and for <5%, it is type D, M, or E; light chain myeloma represent ~15% cases (7).

The causes of MGUS have long remained unknown, although it is established that chronic infection may lead to the emergence of oligoclonal and eventually, monoclonal plasma cells and the subsequent production of a so-called “monoclonal Ig.” In addition, it is thought that certain genetic backgrounds, ethnicity, lipid disorders, and obesity may increase the risk of developing myeloma. Consistent with these observations, the study of Ig genes in malignant plasma cells had revealed restricted heavy-chain variable-region use and highly hypermutated Ig heavy- and light-chain genes, which supports antigen-mediated selection of the malignant clone (8, 9). Recent studies identified several types of antigens that are specifically recognized by monoclonal IgGs, notably lysoglucosylceramide (LGL1) (10, 11), and infectious antigens (12–14). Thus, it has been proposed that chronic simulation by glucolipidic auto-antigens or by infectious antigens may underlie the pathogenesis of subsets of IgG MGUS and myeloma.

Identification of LGL1 as a frequent target of the plasma cell clone in MGUS and in myeloma resulted from the study of patients with Gaucher disease (10, 11). Gaucher disease is a genetic disorder in which a glucocerebroside (or glucosylceramide) accumulates, and MGUS and myeloma are more frequent in Gaucher patients than in the general population. Nair et al. demonstrated that the monoclonal Ig of patients with Gaucher disease frequently target LGL1, a glucosylceramide present in excess in these individuals (10, 11). Moreover, up to a third of monoclonal Igs from patients without Gaucher disease—with sporadic MGUS or myeloma—may also target LGL1, which suggested a link between chronic stimulation by a self-antigen and the development of MGUS and myeloma (10, 11). In parallel, our group investigated whether an abnormal immune response to latent infection may lead to MGUS and eventually, myeloma. We designed a new assay, called the multiplexed infectious antigen micro-array (MIAA), which carries proteins and lysates from nine infectious pathogens, to analyze the specificity of infectious antigen recognition of purified monoclonal IgGs from MGUS or myeloma patients (15). Using the MIAA assay, we found that purified monoclonal IgGs reacted with several infectious pathogens known to cause latent infection. These pathogens include herpesviruses, especially Epstein–Barr virus (EBV), and hepatitis C virus (HCV) (12–15). EBV and HCV are oncogenic viruses associated with solid cancers and B-cell malignancies such as Hodgkin and non-Hodgkin lymphoma, mostly directly via cell infection and transformation (16–19). In contrast, EBV DNA is rarely detected in malignant plasma cells (20). Thus, in MGUS and myeloma with EBV-specific IgG, malignant transformation presumably occurs indirectly, without infection of tumor cells, *via* chronic antigen-driven stimulation of the B-cell receptor (BCR) that has identical heavy and light chain specificity to the secreted IgG. Interestingly, monoclonal IgGs may contribute to the inflammation associated with MGUS and myeloma, since they have a very low level of sialylation of the Fc fragment, a characteristic typically associated with a pro-inflammatory action (21).

In contrast to the monoclonal IgGs of MGUS and myeloma patients, the characteristics and antigenic targets of monoclonal IgAs have not been studied. IgA myeloma is relatively rare and differs from IgG myeloma by a worse prognosis and shorter survival: patients with IgA myeloma are considered more at risk of bone destruction, extra-medullary disease, infection, and hyper-viscosity facilitated by greater polymerization of IgAs compared to IgGs (22–28). In the present study, we were able to collect serum from 35 patients with a monoclonal IgA; 25/35 monoclonal IgAs were successfully separated from other Igs. The specificity of antigenic recognition of the purified monoclonal IgAs was analyzed using the MIAA and an adapted LGL1 assay; the isotype of monoclonal IgAs was also determined, and their degree of sialylation was quantified.

## Materials and Methods

### Patients

We examined 35 patients with a monoclonal IgA (6 MGUS, 29 myeloma). Among those, 6 MGUS and 22 myeloma were diagnosed at the University Hospitals (CHUs) in Tours and Bordeaux (France) over the 2010–2016 period. Samples of blood serum from seven additional patients with IgA myeloma from international cohorts of relapsed myeloma were provided by Novartis (Basel, Switzerland).

### Purification of Monoclonal IgAs and Determination of Isotype

After clotting, blood samples were centrifuged at 2,200 × *g* (4°C), and serum aliquots were frozen. Measurement of Ig concentration, separation of monoclonal IgAs from other Igs, and verification of purity were performed as described previously (12–15, 21). Briefly, the presence of a monoclonal IgA in serum is first established in clinical laboratories, then purification of the monoclonal IgA is performed. The protocol of purification starts with a high resolution agarose gel electrophoresis (SAS-MX high resolution; Helena Biosciences, Gateshead, UK), which allows us to elute the monoclonal Ig from the gel, for elution in PBS. The purity of the monoclonal IgA preparation is then verified by isoelectrofocusing on an agarose gel (pH 3–10) followed by blotting and immunorevelation by an anti-human IgA alpha chain antibody labeled with peroxidase. In some cases, the monoclonal IgA preparation still contains very small amounts of other IgAs (oligo- or poly-clonal), always in very low concentration and not detectable by our techniques. Moreover, eventual contamination by IgG is not relevant here because all further assays are revealed using anti-human IgA alpha chain antibodies.

To determine the A1/A2 isotype, 96-well plates (Nunc MaxiSorp™) were coated overnight at 4°C with 50 μl of goat anti-human IgA antibody (Southern Biotech, Birmingham, AL, USA) diluted 1:500 in 25 mM borate buffer pH 9. After washing with PBS-Tween 0.05%, wells were saturated for 2 h at 37°C with 0.25% B-grade bovine gelatin (Sigma, St. Louis, MO, USA) diluted in 0.1% PBS-Tween; 50 μl of monoclonal IgA (400 ng/ml) was then added (2-h incubation, 37°C). After washing, 50 μl of biotinylated mouse anti-human IgA1 or IgA2 antibody (0.5 μg/ml; Southern Biotech, Birmingham, AL, USA) was added (2-h incubation, 37°C). After washing, 50 μl of streptavidin-HRP (1 μg/ml; Vector Laboratories, Burlingame, CA, USA) was added (1-h incubation, 37°C). After washing, 50 μl of 3,3′,5,5′-tetramethylbenzidine (TMB) was added. The reaction was stopped with 50 μl of sulfuric acid (0.5 M). Optical density was read at 450 nm using a Spark 10 M multimode microplate reader (Tecan, Männedorf, Switzerland).

### The MIAA Assay

The MIAA assay allows testing for panels of commercially available proteins, antigens, or/and lysates from EBV, herpes simplex virus 1 (HSV-1), HSV-2, cytomegalovirus (CMV), varicella zoster virus (VZV), HCV, *Helicobacter pylori* (*H. pylori*), *Toxoplasma gondii* (*T. gondii*), and *Borrelia burgdorferi* (*B. burgdorferi*) (12–15, 21). For incubation on MIAA arrays, Ig concentrations were adjusted to 400 μg/ml (serum) or 50–200 μg/ml (purified monoclonal IgAs) in 80 μl. After washing, MIAA slides were incubated with Dylight^TM^ 680-labeled goat anti-human IgA Fc antibody (1:2,500; 0.4 μg/ml; Immuno Reagents, Raleigh, NC, USA). Fluorescence signals were detected with the Odyssey infrared imaging system scanner at 21-μm resolution (LI-COR Biosciences, Lincoln, NE, USA) and quantified using the GenePix® Pro 4 Microarray Acquisition and Analysis Software (Molecular Devices, Sunnyvale, CA, USA) (12–15, 21). Five fluorescence thresholds of specific positivity were determined using positive and negative controls: 500, for HCV, *H. pylori, T. gondii*; 1,000, for HSV-1 and HSV-2; 1,200, for CMV; 1,400, for EBV and VZV; and 1,800 for *B. burgdorferi*. Fluorescent signals below the thresholds were considered negative (12, 21).

### Dot Blotting Assays

Nitrocellulose membranes (Amersham, Buckinghamshire, UK) were spotted with recombinant EBNA-1 protein, relevant and irrelevant EBV peptides, or HCV core protein, then dried (12). Saturation of membranes, and incubation with serum or purified monoclonal IgA were performed as published (12). Antigen–IgA complexes were revealed using an HRP-labeled goat anti-human IgA (α chain) from Bethyl Laboratories (Montgomery, TX, USA).

### LGL1 Immunoblotting Assay

For LGL1-specific immunoblotting, polyvinylidene fluoride (PVDF) membranes were incubated for 90 min in 100 μg/ml of LGL1 in 0.1 M sodium bicarbonate, rinsed 3 times in PBS and 0.1% Tween 20 detergent, then blocked for 2 h with 5% bovine serum albumin (BSA) (10, 29). Samples of serum or purified monoclonal IgAs were submitted to agarose gel electrophoresis; then, the gels were blotted onto the LGL1-saturated membranes by diffusion blotting during 12 min (10, 30). After blocking for 1 h with 2.5% BSA in PBS and 0.1% Tween 20, membranes were incubated with anti-human IgA horseradish peroxidase (HRP)-conjugated secondary antibody for 1 h, then washed and revealed with Super Signal West Pico chemiluminescent substrate (Thermo Scientific).

### Analysis of the Sialylation of Serum IgAs

An enzyme linked lectin assay (ELLA) was developed to analyze IgA sialylation, and an enzyme linked immuno-sorbent assay (ELISA) was used for the detection of total IgAs, as previously described (21). Ninety-six well plates (Nunc MaxiSorp™) were coated overnight at 4°C with 50 μl of goat anti-human IgA (Bethyl Laboratories, Montgomery, TX, USA) diluted 1:250 (4.0 μg/ml; ELLA) and 1:1,000 (1.0 μg/ml; ELISA) in 25 mM borate buffer pH 9. After three washes with 200 μl of PBS-Tween 0.05% (Sigma, Saint Louis, USA), 100 μl of periodic acid (5 mM) per well was added for 10 min at room temperature, protected from light. The plates were then saturated with 100 μl of 0.25% B-grade bovine gelatin (Sigma, St. Louis, MO, USA) in PBS-Tween 0.01%, at 37°C, for 2 h. After three washes, samples were diluted in PBS-Tween 0.1% and deposited in triplicate wells containing 1.25 ng of Ig for the detection of total IgA, or 2.5 ng of IgA for sialylation studies. The total IgA quantity was assessed by incubating the plates with 50 μl of HRP-coupled goat anti-human IgA diluted 1:1,000 for 1 h (Bethyl Laboratories, Montgomery, TX, USA). Sialic acid was revealed using 50 μl of biotinylated *Sambucus nigra* agglutinin (SNA) diluted 1:750 (2 μg/ml; Glycodiag, Orléans, France) for 90 min and then 50 μl of streptavidin HRP diluted 1:1,000 (1 μg/ml; Vector laboratories, Burlingame, CA, USA) for 1 h, at 37°C. Then, 50 μl of TMB, the chromogenic substrate for HRP (Sigma-Aldrich, St. Louis, MO, USA) was added, and the reaction was stopped by 50 μl of 0.5 M sulfuric acid, after 3 min for IgA detection, and after 5 min for sialic acid detection. Optical densities (OD) were measured using a Spark 10 M multimode microplate reader (Tecan, Männedorf, Switzerland) at 450 nm. The percentage of sialylation was calculated as follows: [SNA OD signal/IgA OD signal]/[ng IgA in SNA well/ng IgA in IgA well] × 100. In all experiments, internal controls were used to assess reproducibility.

### Statistics

Data analysis was performed by GraphPad Prism 6.01 software. Patient parameters were expressed as medians and ranges, or/and means ± standard error of the mean (SEM). The Chi-2 test was used. For continuous variables, a Mann–Whitney *U*-test or a Kruskal–Wallis test followed by Dunn's *post-hoc* test was performed. The tests used are indicated in the legends of figures and tables. A value of *p* < 0.05 was considered statistically significant.

### Study Approval

The study was promoted by the CHU of Nantes, France (# RC12 0085) with the approval of the local and national ethical committee [Comité Consultatif de Protection des Personnes dans la Recherche Biomédicale, Commission Nationale de l'Informatique et des Libertés (CNIL #912335)]. Written informed consents were obtained from patients and healthy donors, by the blood bank (Etablissement Français du Sang (EFS), Nantes, France). A convention was signed between CRCINA and EFS Pays de La Loire.

## Results

### Characteristics of Patients With a Monoclonal IgA

In this retrospective study, 35 patients with a monoclonal IgA were recruited (6 MGUS, 29 myeloma). Annotated clinical data were available for 26 patients (6 MGUS, 20 myeloma); the biological and clinical characteristics of patients are shown in Table 1. The male/female ratios were 33.3% for MGUS and 45.0% for myeloma, and the median age of MGUS and myeloma patients with monoclonal IgA at the time of diagnosis was 76.6 and 75.1, respectively. Thus, in this cohort, patients with IgA myeloma were older than in the series of 135 IgG myeloma patients we studied previously (median age at the time of diagnosis: 75.1 years for IgA myeloma vs. 63.8 years for IgG myeloma) (12, 21). Compared to IgG myeloma, the quantity of monoclonal Ig produced at the time of diagnosis of IgA myeloma was low (median quantity of monoclonal Ig: 17.0 g/L for IgA vs. 26.7 g/L for IgG), and the median percentage of plasma cells in the bone marrow was high: 52 vs. 33% for IgG myeloma (12). All but one patients with IgA myeloma presented with bone lesions, and the International Staging System (ISS) and Durie–Salmon Staging (DSS) scores indicated that 50.0% of patients presented with ISS stage III at the time of diagnosis (median DSS stage III: 59.1%).

**Table 1:** Characteristics of patients with IgA monoclonal gammopathy of undetermined significance (MGUS) or IgA myeloma.

	**MGUS**	**Myeloma**
Patients, *n*	6	29
Patients with available biological data, *n*	6	20
Male sex, *n* (%)	2 (33.3%)	9 (45.0%)
**Age (year)**		
Median	76.6	75.1
Range	66–97	57–95
**Monoclonal IgA (g/L)**		
Median	8.0	17.0
Range	3.0–19.0	4.0–57.0
**Bone marrow plasma cells (%)**	*n* =2	*n* = 19
Median	8	52
Range	1–16	1–89
**β_2_-microglobulin (mg/L)**		
Median	NA	4.2
Range	NA	1.1–14.0
**Leukocytes (×10^9^/L)**		
Median	7.1	4.6
Range	4.1–11.8	1.3–9.0
**Hemoglobin (g/dl)**		
Median	11.2	9.7
Range	7.6–126	5.4–15.3
**Platelets (×10^9^/L)**		
Median	218.5	164.0
Range	132–356	24–269
ISS (*n* = 8)^*^		
Stage I, *n* (%)	-	2 (25.0%)
Stage II, *n* (%)	-	2 (25.0%)
Stage III, *n* (%)	-	4 (50.0%)
**DSS (*n* = 22)**		
Stage I, *n* (%)	-	0 (0%)
Stage II, *n* (%)	-	9 (40.9%)
Stage III, *n* (%)	-	13 (59.1%)

*n, number; NA, not available; ISS, International System Staging; DSS, Durie–Salmon Staging*.

* *The β_2_-microglobulin level was available for only eight myeloma patients*.

### Serological Status of MGUS and Myeloma Patients With a Monoclonal IgA

The unseparated IgG and IgA serological status was determined for 32 patients (6 MGUS, 26 myeloma) using the MIAA, which tests for reactivity to lysates and/or antigens representing nine infectious pathogens (12, 15, 21). The MIAA assay can be revealed either with a fluorescent goat anti-human IgG Fc antibody (for IgG serology) or with a fluorescent goat anti-human IgA Fc antibody (for IgA serology), which allowed us to analyze in parallel IgG and IgA reactivity in the serum of patients (Table 2). Overall, the rates of positive IgG serology for EBV, CMV, HSV-1, *T. gondii* of MGUS, and myeloma patients were comparable to those of our previous studies and to those observed in the general population. The rates of positive IgG serology differed for HSV-2 (high frequency of positivity in the IgA cohort) and VZV (likely underestimated by the MIAA) (12, 15, 21). Patients also had a positive IgA serology for EBV, CMV, HSV-1, as reported for the general population (31–35). The rates of positive IgA serology were significantly lower than the rates observed for IgG serology for EBV, CMV, HSV-1 (*p* < 0.00001, Fisher exact test), and HSV-2 (*p* = 0.0272, Fisher exact test) (Table 2). They were similar to those of IgG for *H. pylori* and *T. gondii*, and increased for VZV.

**Table 2:** IgG and IgA serological status of MGUS and myeloma patients with monoclonal IgA, as determined with the multiplexed infectious antigen micro-array (MIAA) assay.

**Pathogens**	**MGUS**		**Myeloma**		**MGUS and Myeloma**	
	**(*****n*** **=** **6)**		**(*****n*** **=** **26)**		**(*****n*** **=** **32)**	
	**Negative**	**Positive**	**Negative**	**Positive**	**Negative**	**Positive**
**IgG serology**						
EBV, *n* (%)	0	6 (100%)	0	26 (100%)	0	32 (100%)
HCV, *n* (%)	6	0 (0.0%)	25	1 (3.1%)	31	1 (3.1%)
CMV, *n* (%)	4	2 (33.3%)	12	14 (53.8%)	16	16 (50.0%)
HSV-1, *n* (%)	1	5 (83.3%)	7	19 (73.1%)	8	24 (75.0%)
HSV-2, *n* (%)	3	3 (50.0%)	15	11 (42.3%)	18	14 (43.8%)
VZV, *n* (%)	5	1 (16.7%)^*^	22	4 (15.4%)^*^	27	5 (15.6%)^*^
*H. pylori, n* (%)	4	2 (33.3%)	19	7 (26.9%)	23	9 (28.1%)
*T. gondii, n* (%)	5	1 (16.7%)	17	9 (34.6%)	22	10 (31.2%)
*B. burgdorferi, n* (%)	5	1 (16.7%)	26	0 (0.0%)	31	1 (3.1%)
**IgA serology**						
EBV, *n* (%)	2	4 (66.7%)	13	13 (50.0%)	15	17 (53.1%)^a^
HCV, *n* (%)	6	0 (0.0%)	25	1 (3.8%)	31	1 (3.1%)
CMV, *n* (%)	5	1 (16.7%)	26	0 (0.0%)	31	1 (3.1%)^b^
HSV-1, *n* (%)	4	2 (33.3%)	24	2 (7.7%)	28	4 (12.5%)^c^
HSV-2, *n* (%)	5	1 (16.7%)	22	4 (15.4%)	27	5 (15.6%)^d^
VZV, *n* (%)	4	2 (33.3%)	14	12 (46.2%)	18	14 (43.8%)^e^
*H. pylori, n* (%)	5	1 (16.7%)	18	8 (30.8%)	23	9 (28.1%)
*T. gondii, n* (%)	5	1 (16.7%)	23	3 (11.5%)	28	4 (12.5%)^f^
*B. burgdorferi, n* (%)	5	1 (16.7%)	21	5 (19.2%)	26	6 (18.8%)

*Prior to the analysis of the specificity of purified monoclonal IgAs, the IgG serology status and the IgA serology status were assessed in parallel for 32 patients (6 MGUS, 26 myeloma) by analyzing serum samples (containing unseparated IgGs, unseparated IgAs, and monoclonal IgA) with the MIAA assay, revealed with either a Dylight^TM^ 680-labeled goat anti-human IgG Fc antibody or a Dylight^TM^ 680-labeled goat anti-human IgA Fc antibody*.

* *Underestimated by the MIAA assay, the percentage of positive IgG serology for VZV in the general population being >90%*.

a *p < 0.00001*.

b *p < 0.00001*.

c *p < 0.00001*.

d *p = 0.0272*.

e *p = 0.0272*.

f *p = 0.1289 compared to the IgG serology, Fisher exact test*.

*EBV, Epstein–Barr virus; HCV, hepatitis C virus; CMV, cytomegalovirus; HSV, herpes simplex virus 1; VZV, varicella zoster virus; H. pylori, Helicobacter pylori; T. gondii, Toxoplasma gondii; B. burgdorferi, Borrelia burgdorferi*.

### Identification of the Infectious Targets of Purified Monoclonal IgAs

**Figure 1:**
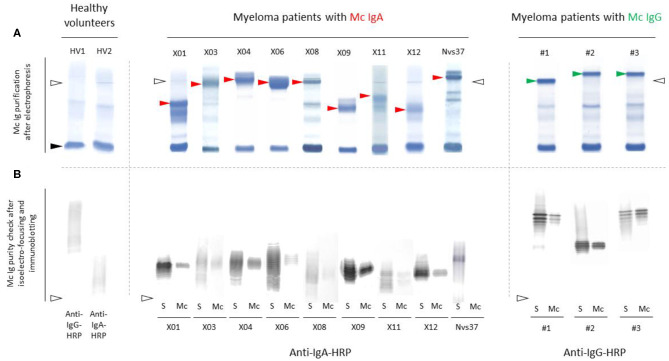
Separation of monoclonal IgAs from other Igs in serum. **(A)** Monoclonal IgAs (red arrowheads) and monoclonal IgGs used as controls (green arrowheads) were separated from other Igs in serum as published (9–11, 19). In the serum of healthy volunteers (HV), only polyclonal Igs are found; thus, no band is seen, only a smear. Note that monoclonal IgAs migrate in the β zone, are less well-separated than monoclonal IgGs, and thus are more difficult to purify than monoclonal IgGs. **(B)** The purity of monoclonal IgAs and IgGs was verified using isoelectric focusing (IEF) and immunoblotting. **(A,B)** Nine examples are shown for monoclonal IgAs and three for monoclonal IgGs (S, serum; Mc, monoclonal Ig separated from polyclonal Igs). Polyclonal Igs are represented by smears, whereas monoclonal Igs are represented by a single band in serum protein electrophoresis **(A)** and due to different migration according to different degrees of sialylation during IEF, as a stack of bands **(B)**. The lines of sample deposit are indicated by white arrowheads. The albumin band is shown with a black arrowhead **(A)**.

**Figure 2:**
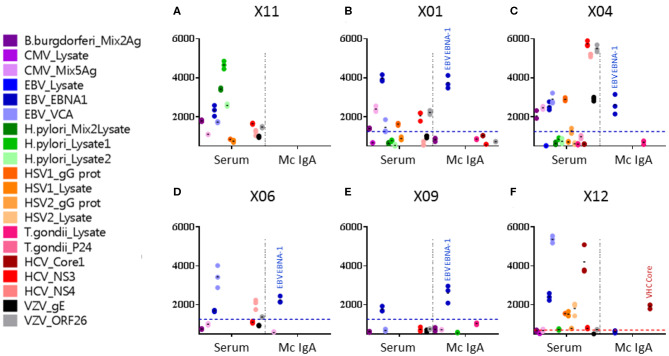
Viral targets of purified monoclonal IgAs from myeloma patients, as determined by the multiplexed infectious antigen micro-array (MIAA) revealed using a Dylight^TM^ 680-labeled goat anti-human IgA Fc antibody. For each patient, serum and purified monoclonal (Mc) IgA were incubated in parallel in the MIAA assay; results shown as fluorescent intensity represent either unseparated IgAs (left) or the patient's monoclonal IgA (right). **(A)** A patient with a Mc IgA that does not react with any pathogen of the MIAA. **(B–E)** Four patients with Epstein–Barr virus (EBV)-specific Mc IgAs. **(F)** One patient with a hepatitis C virus (HCV)-specific Mc IgA. EBV nuclear antigen (EBNA-1) signals are shown in dark blue dots, HCV core signals in red dots, and positive thresholds are shown in dotted lines. **(A)** For patient X11, the serum contained IgAs that recognized *Borrelia burgdorferi*, EBV EBNA-1, EBV VCA, *Helicobacter pylori* lysates 1 and 2, HCV NS3, and varicella zoster virus (VZV) ORF26 protein, whereas the purified Mc IgA did not recognize anything on the MIAA array. **(B)** For patient X01, the serum contained IgAs that recognized a mix of cytomegalovirus (CMV) antigens, EBV EBNA-1, EBV VCA, herpes simplex virus (HSV-1) gG, HCV NS3, and VZV ORF26; the purified Mc IgA recognized EBV EBNA-1 only. **(C)** For patient X04, the serum contained IgAs that recognized *B. burgdorferi*, CMV antigens, EBV EBNA-1, EBV VCA, HSV-1 gG, HCV NS3, HCV NS4, VZV gE, and ORF26, whereas the purified Mc IgA recognized EBV EBNA-1 only. **(D)** For patient X06, the serum contained IgAs that recognized EBV EBNA-1, EBV VCA, and HCV NS3; the purified Mc IgA recognized EBV EBNA-1 only. **(E)** For patient X09, both IgAs in serum and the purified Mc IgA recognized the EBV EBNA-1 protein only. **(F)** For patient X12, the serum contained IgAs that recognized EBV EBNA-1, EBV VCA, HSV-1 gG, HSV-1 lysate, HSV-2 lysate, and HCV core, whereas the purified Mc IgA recognized HCV core only. **(B–F)** The fluorescence values shown for EBV EBNA-1 or HCV core were obtained after subtraction of the non-specific fluorescent background. Thresholds of specific positivity were defined for each viral pathogen or protein (1,400 for EBV EBNA-1, blue threshold; 500 for HCV core, red threshold) (9, 14, 19). Note that dots may be superimposed; horizontal bars represent the means of results obtained for a pathogen, Ag, or lysate. Experiments were performed in triplicates, repeated at least once.

**Figure 3:**
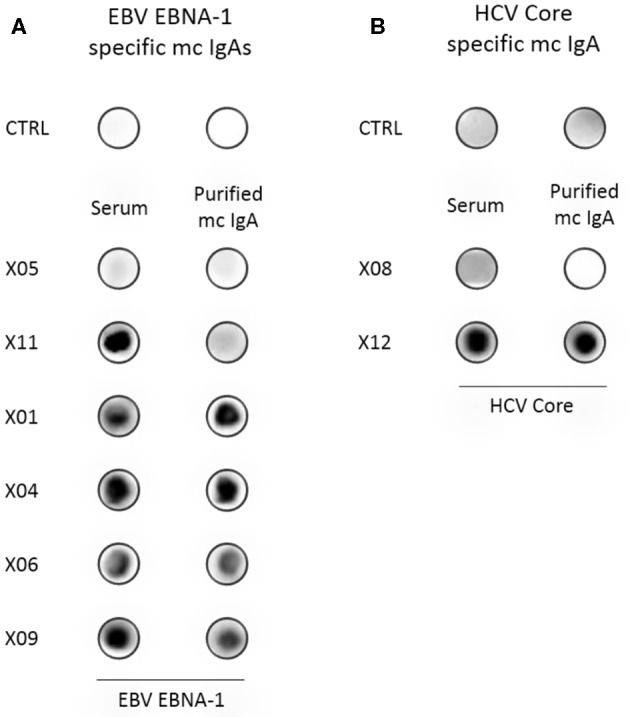
Confirmation of the specificity of recognition of EBV EBNA-1 or HCV core proteins by purified monoclonal IgAs. **(A)** Dot blotting assays with purified recombinant EBNA-1 were performed in parallel with PBS, as control (CTRL), and with the serum and the purified monoclonal IgA from six patients. As assessed by the MIAA array, both serum and purified monoclonal IgAs of the patient X05 did not recognize EBV EBNA-1, and only unseparated IgAs from the serum of the patient X11 recognized EBNA-1 (negative controls). For patients X01, X04, X06, and X09, both serum and purified monoclonal IgAs recognized EBV EBNA-1, thus confirming the results obtained with the MIAA array. **(B)** A dot blotting assay with purified recombinant HCV core protein was performed in parallel with PBS, as control (CTRL), and with the serum and purified monoclonal IgA of patients. As assessed by the MIAA array, both the serum and the purified monoclonal IgA of patient X08 did not recognize the HCV core (negative control). For patient X12, both the serum and the purified monoclonal IgA recognized the HCV core, confirming the results obtained with the MIAA array. Experiments were performed at least twice.

Monoclonal IgAs were separated individually from other Igs from blood serum, then incubated on the MIAA. In general, monoclonal IgAs were more difficult to purify than monoclonal IgGs because of their migration in agarose in the beta–gamma zone (Figure 1). Altogether, purity was achieved for 25 (71.4%) monoclonal IgAs (6 MGUS, 19 myeloma). The A1 or A2 isotype was determined for 21 monoclonal IgAs: 20 were IgA1s (5 MGUS, 15 myeloma) and 1 was an IgA2 (1 MGUS, X01). None of the monoclonal IgAs from MGUS patients recognized any infectious pathogen in the MIAA (Figure S1), whereas the purified monoclonal IgAs from five (26.3%) myeloma patients in this series specifically recognized a single recombinant protein from a single pathogen. Four recognized EBV nuclear antigen-1 (EBNA-1) (Figures 2A–E), and one recognized HCV core protein (Figure 2F). MIAA results of other myeloma patients are shown in Figure S1. Dot blots with recombinant EBNA-1 or HCV core proteins confirmed a positive reaction for the monoclonal IgAs specific for EBNA-1 (Figure 3A) or HCV core (Figure 3B). EBNA-1-specific monoclonal IgAs were then tested against an immuno-dominant B-cell public epitope sequence, PGRRPFF (EBNA-1 residues 400–406), reported to be a target for polyclonal Igs from 86.3% of the general population (36). However, we previously found that the PGRRPFF sequence was recognized by only 2/32 (6.25%) of EBNA-1-specific monoclonal IgGs (12). In this cohort of IgA myelomas, none of the four EBNA-1-specific monoclonal IgAs recognized PGRRPFF (data not shown).

### Identification of Monoclonal IgAs That Target LGL1

**Figure 4:**
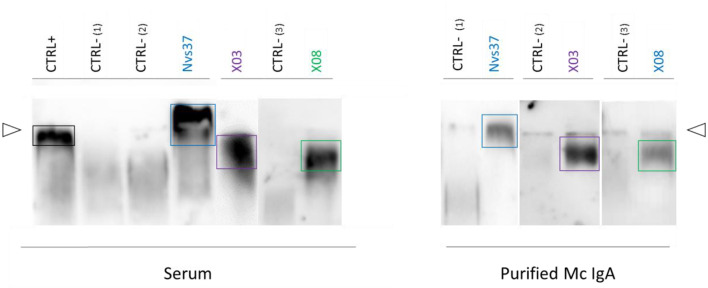
Lysoglucosylceramide (LGL1) is specifically recognized by subsets of purified monoclonal IgAs. LGL1-specific immunoblotting assays were performed as described in the Materials and Methods section (7, 27, 28). Samples of serum (left) or purified monoclonal IgAs (right) were first submitted to agarose gel electrophoresis; then, the gels were blotted onto LGL1-saturated membranes. After blocking for 1 h, membranes were incubated with anti-human IgA horseradish peroxidase (HRP)-conjugated secondary antibody, then washed and revealed by chemiluminescence. The positive control (CTRL+, left) was a sample of serum from a patient known to have LGL1-specific IgAs. Negative controls (CTRL–) were samples of serum without LGL1-reactive IgAs (one from a healthy volunteer, two from patients). The lines of sample deposit are indicated by white arrowheads. The positive signals characteristic of LGL1-reactive Igs are encircled. Patterns of migration may differ for serum and purified monoclonal IgAs because serum may contain both monoclonal and polyclonal LGL1-reactive IgAs.

Purified monoclonal IgAs from MGUS and myeloma patients were analyzed with the LGL1 assay adapted from Nair et al. (10). As shown in Figure 4, only three monoclonal IgAs reacted with LGL1, and all were from myeloma patients (X03, X08, Nvs37). The monoclonal IgA of each of the three patients did not recognize any pathogen of the MIAA assay (their MIAA assay was negative, an indirect proof of purity of the monoclonal IgA preparation). Thus, 3/19 (15.8%) of myeloma monoclonal IgA targeted LGL1 in this series.

### Characteristics of Myeloma Patients With a Monoclonal IgA Specific for EBNA1 or LGL1

We compared the clinical characteristics of the four myeloma patients with EBNA-1-specific monoclonal IgA [“EBNA-1(+)” patients] with those of the 17 myeloma patients with a monoclonal IgA that was non-reactive for pathogens of the MIAA assay [“MIAA(–)” patients] (Table 3). Compared to other IgA myeloma patients, myeloma patients with EBNA-1-specific IgA were relatively young at the time of diagnosis (≤63 years old), as reported for myeloma patients with EBNA-1-specific monoclonal IgG (12). Regarding patients with an LGL1-specific monoclonal IgA, Nair et al. reported a tendency toward a mild form of disease for LGL1-associated myeloma (10). Here, clinical and biological characteristics were available for only two patients. The data did not suggest a mild disease since both had bone lesions: one had >50% plasma cells in the bone marrow, and one had a DSS stage III (Table 4).

**Table 3:** Characteristics of myeloma patients with a monoclonal IgA specific for EBV nuclear antigen 1 (EBNA-1).

	**Myeloma with or without a monoclonal IgA specific for EBV or HCV**			
	**HCV(+)**	**EBNA-1(+)**	**MIAA(–)**	***p*-value**
**Patients**, *n*	1	4	17	
Male sex, *n* (%)	1	0 (0%)	7 (41.2%)	
**Age at Diagnosis** (year)				
Patients, *n*	1	3	17	
Median	NA	60.6	75.1	
Mean ± SD	NA	60.2 ± 2.9	73.5 ± 8.8	*p* = 0.0254^*^
Range, min–max	94	57–63	57.1–86.5	
**Leukocytes** (10^9^/L)				
Patients, *n*	1	3	16	
Median	NA	3.6	5.1	*NS*
Range, min–max	NA	3.4–4.6	1.3–9.0	
**Hemoglobin** (g/dl)				
Patients, *n*	1	3	16	
Median	NA	9.8	9.7	*NS*
Range, min-max	NA	8.5–12.5	5.4–15.3	
**Platelets** (10^9^/L)				
Patients, *n*	1	3	16	
Median	NA	140	169	*NS*
Range, min–max	NA	113–237	24–269	
**Bone Marrow Plasma Cells** (%)				
Patients, *n*	1	3	16	
Median	NA	74	46.5	*p* = 0.0572^*^
Range, min–max	NA	59–93	1^#^-89	
**Calcemia** (mmol/L)				
Patients, *n*	1	3	17	
Median	NA	2.4	2.2	*NS*
Range, min–max	NA	2.3–2.6	1.8–2.7	
**Creatinin** (μmol/L)				
Patients, *n*	1	3	17	
Median	NA	59.0	69.0	*NS*
Range, min-max	NA	57–117	36–763	
**β**_**2**_-**Microgobulin** (mg/L)				
Patients, *n*	1	2	5	
Median	NA	5.6	4.5	*NS*
>3.5 mg/L, *n* (%)	NA	1 (50.0%)	2 (40.0%)	
Range, min–max		3.05–8.2	1.1–14.2	
**Monoclonal IgA** (g/L)				
Patients, *n*	1	4	17	
Median	NA	33.0	16.0	*NS*
Range, min-max	NA	17.0–57.0	4.0–57.0	
**Bone Lesions**				
Patients, *n*	1	3	17	
With bone lesions, *n* (%)	NA	3 (100%)	15 (88.2%)	*NS*
**DSS Stage**				
Patients, *n*	1	4	17	
Stage I	NA	0	0	
Stage II	NA	1	7	
Stage III, *n* (%)	NA	3 (75.0%)	10 (58.8%)	*NS*
**ISS Stage**				
Patients, *n*	1	4	5	
Stage I, *n*	NA	2	1	
Stage II, *n*	NA	0	2	
Stage III, *n* (%)	NA	2 (50.0%)	2 (40.0%)	*NS*
**Serum IgA Sialylation (%)**				
Patients, *n*	1	3	17	
Median (mean)	49.7 (49.7)	53.4 (41.7)	40.8 (43.1)	
Range, min–max	NA	11.9–59.7	35.9–57.9	*NS*

n, number; NA, not applicable; NS, not significant; HCV+, patient with HCV-specific purified monoclonal IgA; EBNA-1+, patients with EBNA-1-specific purified monoclonal IgA; MIAA-, Patients with a purified monoclonal IgA not specific for any infectious pathogen of the MIAA. Because biological information was not available for all patients, and was partial for some patients, the number of patients with data may vary. Statistical analysis was performed using the Chi-2 test for categorical variables and the

* *Mann–Whitney test for continuous variables. Significant differences are indicated*.

# *MM patient with 29g/L monoclonal IgG*.

**Table 4:** Characteristics of myeloma patients with a lysoglucosylceramide (LGL1)-specific monoclonal IgA.

	**Patient X03**	**Patient X08**	**Patient Nvs37**
Sex (M/F)	F	F	M
Age (year)	76	66	51
Leukocytes (×10^9^/L)	2.4	7.2	NA
Hemoglobin (g/dl)	6.9	12.8	NA
Platelets (×10^9^/L)	191	269	NA
Bone marrow plasma cells (%)	51	16	NA
Calcemia (mmol/L)	2.16	2.36	NA
Creatinin (μmol/L)	66	38	NA
β_2_-microglobulin (mg/L)	NA	NA	NA
Monoclonal IgA (g/L)	28.0	8.0	23.2
Bone lesions	Yes	Yes	NA
ISS stage	NA	NA	NA
DSS stage	NA	III	NA
Serum IgA sialyation (%)	36.1%	46.3%	NA

*NA, not available; ISS, International System Staging; DSS, Durie–Salmon Staging*.

### Glycosylation of IgAs

**Figure 5:**
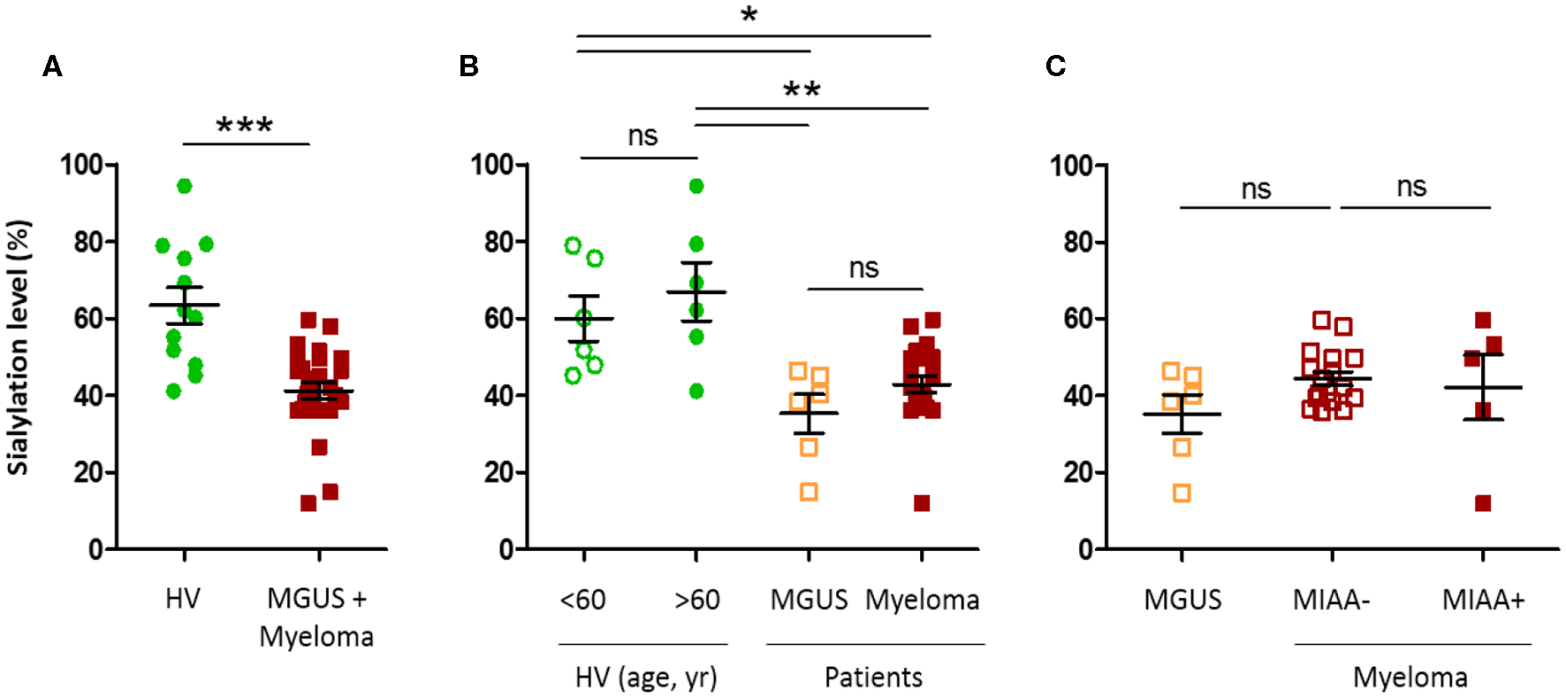
Hyposialylation of IgAs from monoclonal gammopathy of undetermined significance (MGUS) and myeloma patients. The sialylation level of IgAs in the serum of healthy volunteers (HV, *n* = 12), MGUS (*n* = 6), and myeloma (*n* = 22) patients was assessed using an enzyme-linked lectin assay (ELLA) technique, as described in the Materials and Methods section. Unfortunately, we were not able to constitute a control cohort of patients with excessive amounts of non-clonal IgAs. Results are expressed as percentages of sialylated forms of IgAs in serum. **(A)** Sialylation level of IgAs from HV (green dots) and MGUS and myeloma (MM) patients (red dots); ***p < 0.001, Mann–Whitney *U*-test. **(B)** Sialylation level according to age, in HV under or over 60 (green dots), and according to the diagnosis of MGUS (orange squares) or MM (red squares). **(C)** Sialylation level of IgAs from MM patients with a pathogen-specific monoclonal IgA, as determined by the MIAA (filled red squares, MIAA+) and MM patients with a monoclonal IgA of undetermined specificity (open red squares, MIAA–), compared to MGUS patients (orange squares). Bars indicate means±SEM. **(B,C)** Significant differences are indicated by stars; *p < 0.05 and **p < 0.01, Kruskal–Wallis test followed by Dunn's *post-hoc* test (ns, not significant).

As published for monoclonal IgGs, the sialylation level of unseparated, total IgAs, assessed in serum, expressed in % sialylation, was lower for MGUS and myeloma patients with a monoclonal IgA than for healthy volunteers (41.2 vs. 63.4%, respectively; ^***^p < 0.0001, Mann–Whitney *U*-test) (Figure 5A) (12, 21). No difference was observed according to age (healthy donors under or over 60 years), nor between MGUS and myeloma (Figure 5B). In addition, there was no difference in IgA sialylation depending on the antigenic specificity (MIAA+ vs. MIAA–) of the monoclonal IgA (Figure 5C, Table 4). However, due to the small size of the cohorts, these results will need to be confirmed in larger studies.

## Discussion

This is the first analysis of the antigenic origins of IgA myeloma. In this small cohort, no antigenic target was identified for the monoclonal IgAs from MGUS patients, but for patients with IgA myeloma, two viruses (EBV, HCV) were candidate targets for the monoclonal IgA of 5/19 patients (26.3%). In addition, the monoclonal IgA of 3/19 myeloma patients (15.8%) reacted with LGL1, a glucolipidic auto-antigen. These results imply that chronic stimulation by viral antigens or auto-antigen LGL1 may underlie the initiation of ~40% of IgA myeloma, as reported for MGUS and myeloma with a monoclonal IgG (10, 12).

In this study, myeloma patients with a monoclonal IgA were typically older and had stage III disease, consistent with IgA myeloma being more severe than IgG myeloma (22–28). Analysis of the IgG and IgA serology status of MGUS and myeloma patients revealed that despite stage III disease, patients maintained detectable levels of polyclonal IgGs and IgAs directed against common pathogens. The rates of positive IgG serology against EBV, CMV, HSV-1, and HSV-2 observed for IgA myeloma patients were similar to those of the general population. The rates of positive IgA serology of these patients were lower than those of IgG for EBV, CMV, HSV-1, and HSV-2, as expected (31–35). They were similar to the rates of positive IgG serology for *H. pylori, T. gondii, B. burgdorferi*, and higher for VZV. Thus, this cohort of patients with IgA myeloma maintained IgG- and/or IgA-mediated protection against EBV, CMV, HSV-1, HSV-2, and VZV.

Two viral proteins, EBV EBNA-1 and HCV core, were specifically recognized by monoclonal IgAs from certain myeloma patients; these proteins are also the targets of monoclonal IgGs in both MGUS and myeloma (12–15). Because IgAs are linked with the digestive tract and other mucosal tissues, it is not surprising that monoclonal IgAs reacted with pathogens found in mucosal or digestive and hepatic tissues (HCV) or in saliva (EBV) (31, 35, 37). The PGRRPFF sequence, identified as a frequent EBNA-1 epitope for the general population, was not recognized by EBNA-1-reactive monoclonal IgAs, an observation also made for EBNA-1-reactive monoclonal IgGs (12, 36). It will be important to determine whether the amino acid sequences targeted by monoclonal Igs from myeloma patients differ from those of Igs from healthy individuals. Future characterization of the EBNA-1 sequences recognized by monoclonal IgGs and IgAs should help determine whether certain epitope “hotspots” are overrepresented in the BCR specificity of malignant B cells in myeloma. For instance, knowing the viral sequences linked to MGUS and myeloma would permit their elimination from the future EBV vaccines in development (38, 39). In addition, for 15.8% of patients with IgA myeloma in this cohort, the purified monoclonal IgA reacted with LGL1, a self-antigen initially described as a target of monoclonal Igs in the context of Gaucher disease (10, 11). In future studies, it would be of interest to determine whether MGUS and myeloma patients with an anti-LGL1 monoclonal Ig present a mild, unsuspected metabolic deficiency resulting in sphingolipid accumulation.

Altogether, our findings suggest that for a significant fraction (>30%) of patients with IgA or IgG myeloma, the initial cause of disease may be chronic antigen stimulation due to a viral infection (particularly by EBV or HCV) or autoimmunity against LGL1 (10–14). These observations may be compared to the ~50% chronic lymphocytic leukemia (CLL) where the malignant clone displays somatically mutated Ig heavy (H) chain variable (IGHV) genes, indicative of antigen-driven disease (40, 41). Importantly, antigen-driven disease may be associated with a distinct prognosis: patients with antigen-driven CLL seem to have a more favorable clinical course than other CLL patients, whereas myeloma patients with EBNA-1-associated myeloma tend to present with more severe disease (12, 42, 43). In this small cohort, myeloma patients with EBNA-1-specific monoclonal IgA were relatively young at diagnosis (≤63 years), with severe (59–93%) plasma cell infiltration of the bone marrow, characteristics similar to those reported for myeloma patients with EBNA-1-specific monoclonal IgG (12). Regarding LGL1-associated myeloma, Nair et al. suggested that it may represent a mild form of myeloma (10). In the present study, the two myeloma patients with LGL1-specific IgA did not have mild disease. Studies performed on large cohorts of well-annotated patients (with cytometry, cytogenetics, genetic data) are necessary for the full characterization of myeloma linked to LGL-1 or EBV EBNA-1.

The antigenic targets of malignant clones of B-cell lineage have been studied in the context of CLL, using different technical approaches (phage-display technology, mass spectrometry). Several auto-antigens have been associated with CLL, notably cytoskeleton components (non-muscle myosin heavy chain IIA, vimentin, cofilin-1, filamin B), cardiolipin, proline-rich acidic protein-1 (PRAP-1), dUTPase, and auto-antigens at the surface of apoptotic cells and bacteria (*Streptococcus pneumoniae* for instance) (44–51). Evidence of virus (HCV, HIV)-driven CLL has been less reported (52–54). Regarding EBV, EBV DNA is typically not detected in malignant CLL or myeloma cells (20). The variability of EBV DNA loads in blood and the patterns of anti-EBV Ig responses of patients have been well-analyzed in CLL, but the findings of these studies appear to mostly reflect the deficient immune system of aged CLL patients (55–58). Thus, formal evidence of EBV antigen-driven CLL disease is still lacking, and identified CLL-associated antigens are predominantly auto-antigens linked to bacterial infection and/or apoptotic cell removal, and to a lesser degree, viral antigens (44, 45, 52–54). In contrast, the most frequent antigenic targets associated so far with MGUS and myeloma are viral proteins (especially from EBV, HSV, HCV) and a ganglioside, LGL1 (10–14). Of note, several groups reported that gangliosides facilitate cell entry of viruses (59). Knowing whether anti-LGL1 monoclonal Igs can counter virus cell entry would be of interest.

IgA glycosylation was also analyzed. Monoclonal IgAs differed from IgAs from healthy donors by their low level of sialylation, a characteristic observed for monoclonal IgGs and associated with a pro-inflammatory action of the Ig Fc fragment upon binding to FcγR, notably in monocytes and macrophages (21). However, sialylation did not differ depending on the antigenic specificity of the monoclonal IgA. Further studies are needed to determine whether monoclonal IgAs from MGUS and myeloma patients contribute to the production of pro-inflammatory cytokines, as reported for patients with hyposialylated IgGs (polyclonal or/and monoclonal) (21).

In aging populations, the incidence of MGUS and the subsequent risk of myeloma and other MGUS-associated diseases increase (60, 61). The detection of antigen-initiated MGUS and myeloma cases, and the determination of the antigenic target of the monoclonal Ig, should be useful additions to the diagnostic work-up of MGUS and myeloma because they allow new possibilities of prevention and treatment. First, disease-initiating antigenic targets could serve as new risk markers. Second, MGUS patients, who are not treated presently, and myeloma patients could benefit from antigen-reduction treatments. Supporting this approach, several groups reported that the addition of anti-viral treatment to myeloma protocols resulted in disease regression and/or improved response to chemotherapy, notably for HCV-associated myeloma (62, 63). Drugs that target BCR signaling may also be considered (64, 65). Moreover, new drugs are currently being developed that specifically target EBV (66, 67). Clearly, if one could clear the MGUS-associated underlying chronic infection early on, it may be possible to prevent the development of myeloma (68). Regarding myeloma patients with an LGL1-specific monoclonal Ig, reduction of LGL1 levels may be envisioned as a complementary treatment. Indeed, LGL1 reduction has been successfully achieved in Gaucher patients for many years (69–73). Importantly, glucolipid reduction prevents associated B-cell malignancies in murine models (74, 75). Recently, Nair et al. reported that glucolipid reduction treatment resulted in decreased amount of monoclonal Ig in Gaucher patients with monoclonal gammopathy (76).

In conclusion, EBV EBNA-1, the HCV core protein, and LGL1, a glucolipid, were identified as candidate antigenic targets of the purified monoclonal Igs of patients with IgA myeloma. An abnormal immune response to these viruses or to LGL1 may therefore be part of the pathogenesis of IgA myeloma, as reported for IgG myeloma. Detecting patients who present with LGL1- or virus-associated MGUS or myeloma is important since it is possible to add antigen target reduction to classic treatments.

## Data Availability Statement

The datasets generated for this study are available on request to the corresponding author.

## Ethics Statement

The studies involving human participants were reviewed and approved by Local ethics committee, CHU Nantes. The patients/participants provided their written informed consent to participate in this study.

## Author Contributions

SH, AB, EB-C, and JH designed the research, analyzed the data, and wrote the manuscript. AB, CS, NM, SA-M, MF, EB-C, and JH performed the experiments and edited the manuscript. AT, EP, PL, and FM contributed patient samples and data. All approved the version to be submitted for publication and agreed to be accountable for all aspects of the work in ensuring that questions related to the accuracy or integrity of any part of the article are appropriately investigated and resolved.

## Conflict of Interest

The authors declare that this study received funding from Janssen (USA). Janssen had no role in study design, data collection and analysis, or preparation of the manuscript.
