# Supplementary material for: Analysis of the Targets and Glycosylation of Monoclonal IgAs From MGUS and Myeloma Patients
Source: Front Immunol. 2020 May 27;11:854. doi: 10.3389/fimmu.2020.00854 (PMC7266999; doi:10.3389/fimmu.2020.00854)
Supplement: Supplementary file 1 [file Data_Sheet_1.pdf]

SUPPLEMENTARY MATERIAL

Supplementary Figure 1. ***MIAA results obtained with serum and purified monoclonal IgAs from MGUS and myeloma patients whose monoclonal IgA did not recognize any infectious pathogen of the MIAA assay, revealed using a Dylight™ 680-labelled goat anti-human IgA Fc antibody.***

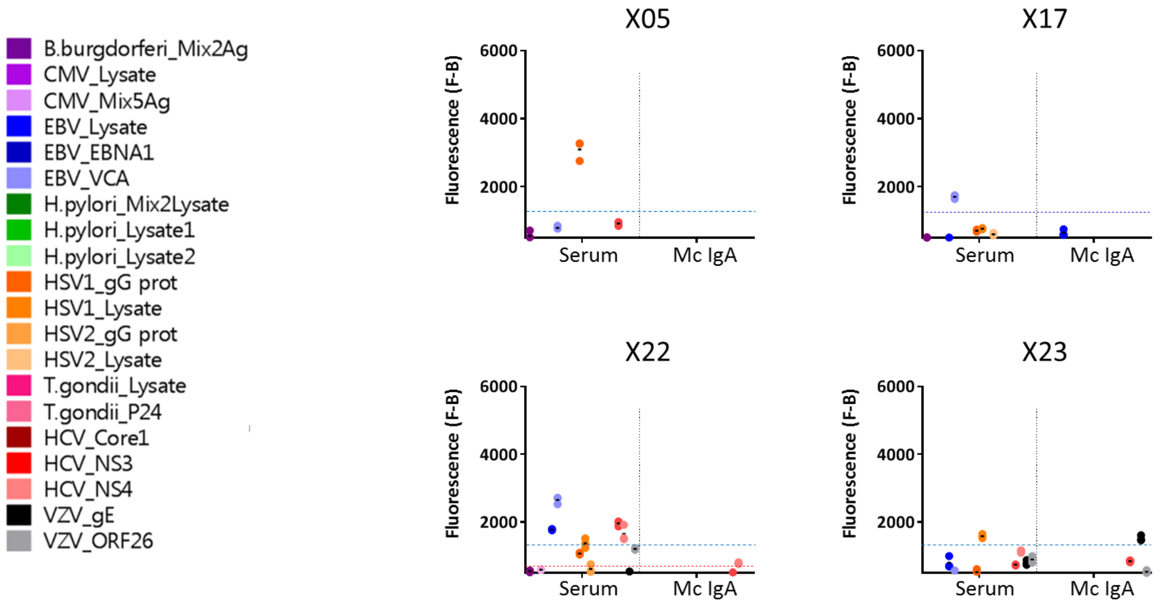

Supplementary Figure 1A. ***MIAA results of 4 MGUS patients whose purified monoclonal IgA did not recognize any pathogen of the assay.*** For each patient, samples of serum and of purified monoclonal (Mc) IgA were incubated in parallel; results shown as fluorescent intensity indicate the different pathogens recognized by unseparated IgAs in serum, whereas the patient's Mc IgA did not recognize any infectious lysate, protein, or antigen in the MIAA. Thresholds of specific positivity are shown in dotted lines (EBV: 1400, blue threshold; HCV: 500, red threshold). Experiments were performed in triplicates, repeated at least once.

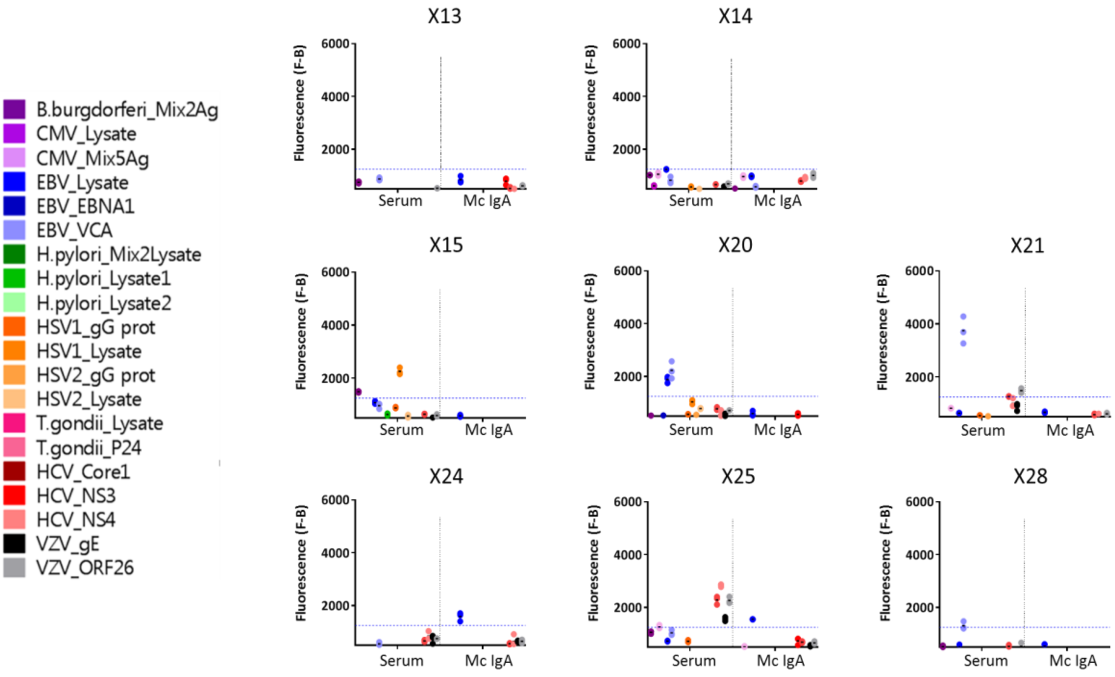

Supplementary Figure 1B. ***MIAA results of 8 myeloma patients whose purified monoclonal IgA did not recognize any pathogen of the assay.*** For each patient, serum and purified Mc IgA were incubated in parallel; results shown as fluorescent intensity indicate the different pathogens recognized by unseparated IgAs in serum, whereas the patient's Mc IgA did not recognize any infectious lysate, protein, or antigen in the MIAA. The EBV EBNA-1 threshold of specific positivity (1400) is shown as a blue dotted line. Experiments were performed in triplicates, repeated at least once. Note that the Mc IgAs of patients X24 and X25 each gave a signal for EBV EBNA-1 at the limit of specificity; the two mc IgAs were tested against recombinant EBNA-1 protein by dot blot assay, with negative results. Therefore, the Mc IgAs from patients X24 and X25 were considered as not recognizing any pathogen of the MIAA assay.

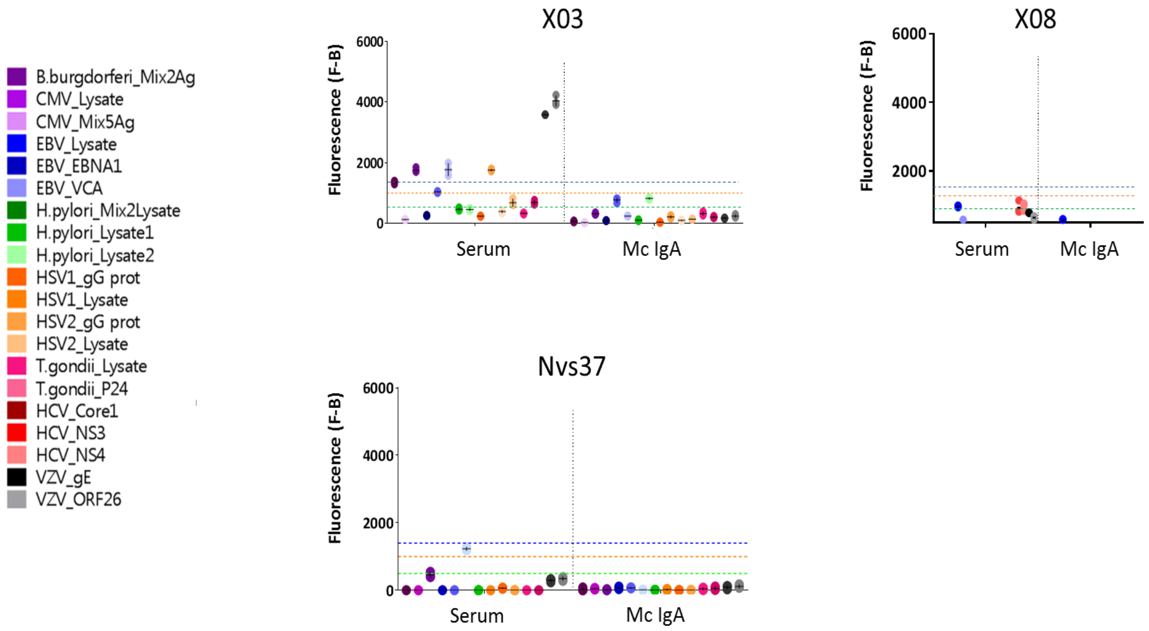

Supplementary Figure 1C. ***MIAA results of the 3 myeloma patients with a LGL1-specific monoclonal IgA.*** For each patient, samples of serum and of purified Mc IgA were incubated in parallel; results shown as fluorescent intensity indicate the pathogens recognized by unseparated IgAs in serum. The three Mc IgAs did not recognize any infectious lysate, protein, or antigen in the MIAA. Thresholds of specific positivity are shown in dotted lines (EBV and VZV: 1400, blue threshold; HSV-1, HSV-2 and CMV: 1000, orange threshold; HCV, *T. gondii* and *H. pylori:* 500, green threshold). Experiments were performed in triplicates.

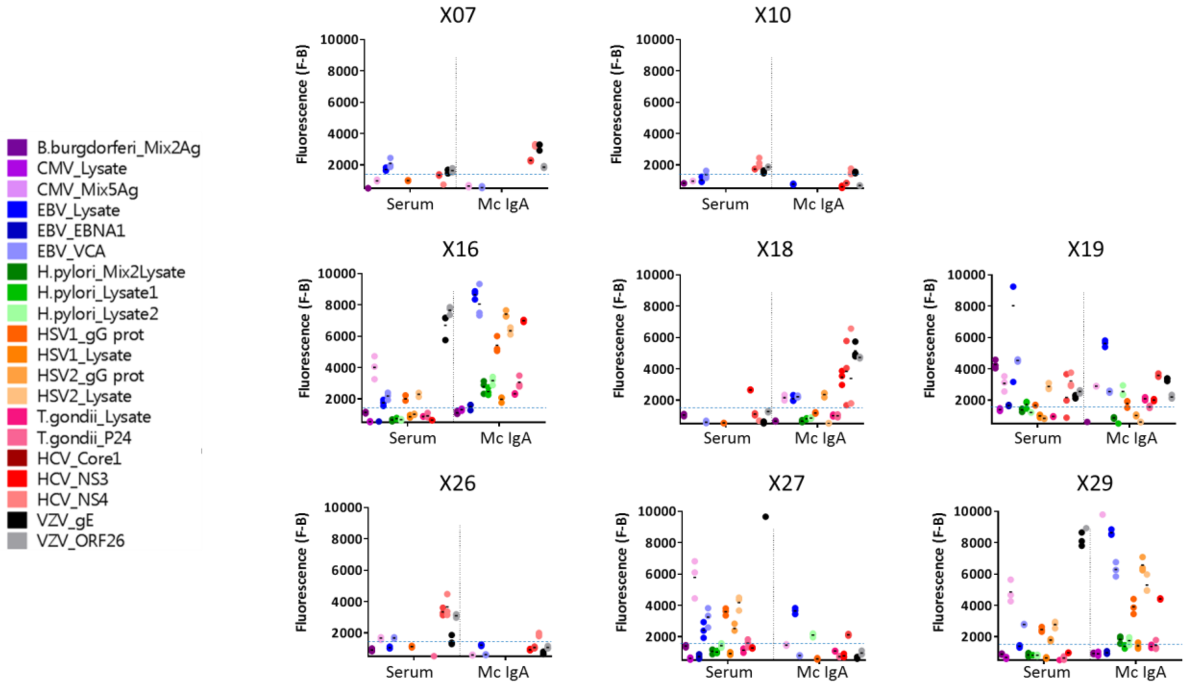

Supplementary Figure 1D. ***MIAA results of 2 MGUS and 6 myeloma patients whose monoclonal IgA could not be purified.*** For each patient, samples of serum and purified Mc IgA were incubated in parallel; results shown as fluorescent intensity indicate the different pathogens recognized by unseparated IgAs in serum. For these 8 patients, the Mc IgA preparation also recognized more than one infectious pathogen. This was expected for the 7 patients (X07, X19, X16, X18, X20, X27, X29) for whom the IEF showed that purification of the Mc IgA was not achieved. For patient X10, the IEF indicated adequate purification of the Mc IgA; the low MIAA signals seen with the purified Mc IgA may be explained by non-specific binding of the Mc IgA, and results were considered inconclusive. For patient X18, MIAA signals obtained for the purified Mc IgA were stronger than those observed for serum, which is most likely due to non-specific binding of the Mc IgA. The EBV EBNA-1 threshold of specific positivity (1400) is shown as a blue dotted line. Experiments were performed in triplicates.
